# Assessing the effects of a 660 nm diode laser on crustacean eyes

**DOI:** 10.1371/journal.pone.0317706

**Published:** 2025-05-06

**Authors:** Rhys A. C. Hague, James E. V. Rimmer, Mark A. James

**Affiliations:** 1 School of Biology, University of St Andrews, St Andrews, United Kingdom; 2 Department of Mathematics, University of York, York, United Kingdom; University of California Santa Barbara, UNITED STATES OF AMERICA

## Abstract

Sustainable management of crustacean fisheries requires accurate and timely data for population modelling, but many stocks are data deficient. To address this challenge, a novel device using Class 3R 660 nm diode lasers and Artificial Intelligence algorithms for automated data collection is under development. Whilst the safe use of Class 3R lasers is prescribed for the human eye, equivalent knowledge is required to ensure that lasers of this Class can be used without causing ocular damage to crustaceans. Some countries recognise crustaceans as sentient, thus ocular impacts that could compromise welfare and impair the subsequent survival of sampled specimens could be deemed unacceptable. This study investigates the impact of a prototype laser scanning system on the compound eyes of the white-legged prawn, *Litopenaeus vannamei*. Histological analysis in a controlled laboratory revealed a correlation between laser exposure and markers of ocular tissue damage, suggesting potential cumulative effects associated with repeated exposure. However, there was indication of pre-existing, underlying baseline alterations in some markers, possibly associated with senescence. Further, observations indicated minimal immediate behavioural effects following single scans, though care is warranted in extrapolating these findings to natural populations and different species under commercial conditions. In an operational context, specimens would only be subjected to a single exposure with a conveyor speed four times faster than that used experimentally, which equates to ~0.05 mW total laser energy. The estimated exposure for a single scan used experimentally was ~0.19 mW. At this level, there is no clear evidence of ocular tissue damage. However, fewer than five repeated exposures at the 6.7 cm/s conveyor speed used experimentally, may result in observable changes in some ocular tissue underscoring the need for cautious protocol development. This research highlights potential biological markers for evaluating photothermal damage in crustacean eyes, which could be used in future studies covering a broader range of commercially significant species.

## Introduction

Sustainable management of fisheries relies on accurate and recent population data [1,2]. Stock assessments of commercially fished crustaceans rely upon the collection of abundance and morphometric data from a combination of fisheries-dependent (market surveys) and fisheries-independent, statistically controlled, at-sea surveys. The logistical challenges and cost of undertaking these surveys limits their scope and frequency to the extent that many stocks are formally “data deficient.” Recent evidence, based on fisheries-dependent surveys alone, concluded that most Scottish crab and lobster stocks are fished at or above maximum sustainable yield [3,4]. Most morphometric data collected for stock assessment purposes is still manually measured and recorded. A novel device for the automated collection of species, sex, and size data from crustaceans is being developed. It uses Class 3R lasers to generate 3D point cloud scan data, which is then analysed using Artificial Intelligence (AI) algorithms to identify the species and the morphometrics used to determine sex and the measurements (+/- 1 mm) for stock assessment purposes. The device is portable for use aboard 10 m fishing vessels, and in land-based holding or processing facilities. It is anticipated that the device would be used as part of a statistically controlled sampling regime in which fishers play an active role in data collection.

Crustaceans are now considered sentient in some countries, including the UK [5], which has implications for the use of any device which could cause them injury. Whilst 3R lasers are considered “safe” with respect to humans operating the lasers, these safety considerations include the assumption that the human blink reflex which takes place in ~0.25 s is sufficient to prevent eye injury in the context of incidental exposure to the laser beam. However, crustaceans lack a blink reflex and are likely more susceptible to laser damage. Lasers of these wavelengths and similar (red light, 620–740 nm) are considered to have a range of effects on organisms, such as toxicity to human fibroblasts, and reducing the amount of reactive oxygen species in human keratinocytes [6]. Red light wavelengths are widely used in medical settings to treat diseases due to evidence of impacting mitochondrial function [7,8].

**Fig 1:**
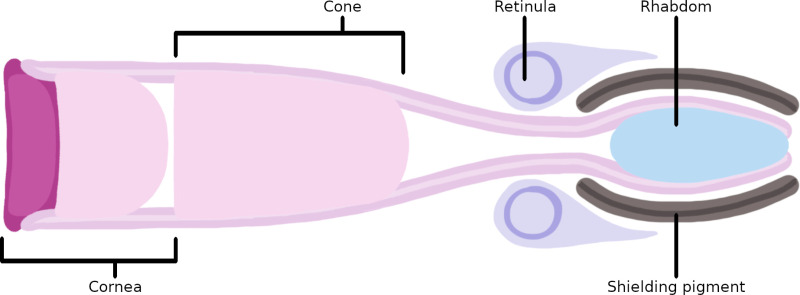
A simplified schematic of a crustacean ommatidium.

The crustacean eye is a compound eye, with decapods possessing the reflecting superposition eye type [9] (Fig 1). This eye type consists of many ommatidia, which have a photo-directive layer and a photoreceptive layer [9,10]. The photo-directive layer consists of a corneal lens which reflects light inwards along the ommatidial cone towards the photoreceptors [11]. The photoreceptive layer consists of multiple photoreceptive retinula cells arranged in a circle around the light-guiding rhabdom in a cross-section, with the orientation of the photoreceptors affecting the polarisation of light they are sensitive to [11,12]. The ommatidia change shape when exposed to light, with the hexagonally arranged microvilli capable of separating outwards and changing in length [13], which is the manner light stimuli is converted into an electrical impulse which can be interpreted by the organism’s brain. The rhabdom itself passes through the basement membrane at the back of the eye towards the optic nerve (Fig 1).

The photo-conductive cornea and cone direct light inwards towards the photoreceptive structure. The photoreceptive structure is visible at the right, with the retinula cells surrounding the photoconductive rhabdom, based on Bryceson and MacIntyre [11].

Crustacean eyes of this type have been shown to be damaged by photic overexposure [10]. The intensity of photic exposure which induces damage can vary between species and populations, with some dark-adapted populations of animals being blinded at ambient sunlight levels. This has been observed in deep-dwelling *Nephrops norvegicus* [14–16], as well as *Cirolana borealis* [13]*, Meganyctiphanes norvegica,* and *Rimicaris exoculata* [15]. Damage is reduced in individuals that have had time to acclimatise to increased light levels by the migration of shielding pigments to the front of the eye [16,17], but it may still occur. Ecological differences between populations, such as residence depth, may cause variation in pigment distribution, and this may be the difference between an organism avoiding damage or sustaining it [16]. As such, the likelihood and severity of damage from the same photic source is context-dependent.

The ways in which photic damage appears to an observer are not well described and most documentation is either written (as in Shelton et al. [16]) or demonstrated only with transmission electron microscopy (TEM) (as in Meyer-Rochow [10]). Markers to quantify the laser impact on the eye were selected from cases of idiopathic blindness, as these are well-documented through light microscopy and written descriptions from prior research.

Descriptions of photic-induced damage include distortion, swelling, and fusion of ommatidial components [17]. Shielding pigments have been suggested to be the epicentre of photothermal damage in the vertebrate eye due to absorbing incoming energy, though damage spreads outwards [18]; this may be true of the crustacean eye as well. In addition, absorptive pigments themselves may be destroyed if subjected to sufficiently high energy absorption [19]. These descriptions suggest visible changes in the morphology of light-blinded eyes, for example, the loss of shielding pigments or the distortion of the ommatidium near pigments.

Idiopathic blindness markers are taken from cases of blindness in *Homarus americanus* as described by Maniscalco and Shields [20], Magel et al. [21], and Shields et al. [22]. The damage associated with these markers is likely not caused by light exposure [20] but some may still manifest in cases of photothermal damage. The first potential marker of photic damage is the clumping of shielding pigments. Additionally, damaged rhabdom may appear “ragged” rather than “spindle-shaped” and the optic nerve fibres behind the rhabdom may become necrotic. The basement membrane between the nerve fibres and the rhabdom ruptures, allowing infiltration of haemocytes into the eye. Lesions in the eye are also visible in some cases of blindness.

The markers selected for quantification in this study include gross ommatidial and basement membrane alteration, melanin “clumping,” melanin-associated tissue loss, haemocyte infiltration, lesion presence, and optic nerve fibre necrosis. These markers were quantified in haematoxylin and eosin (HE) stained tissue. The white-legged prawn *Litopenaeus vannamei* (“prawn”) was selected as a case study. This study aims to investigate the impact of a prototype laser scanner, which has been developed for crustacean fisheries research, on the decapod eye.

## Materials and methods

### Animal acquisition and care

The prawns were acquired from, cultured and cared for by RASTECH Research CIC. RASTECH Research CIC cared for the prawns following methodology from Treece and Fox [23]. Twenty-nine *L. vannamei* individuals, estimated to be approximately two years of age based on body length, were housed together in a 5 m^3^ cylindrical tank in a water depth of 1 m. The light in the tank room was ~24 lux at a wavelength of 480 nm. There was no evidence of overcrowding or starvation in the studied animals. The enclosure was filled with oxygenated seawater, which was maintained at 28 °C and a salinity of 34 ppm, ammonia 0 ppm, nitrite 0 ppm, nitrate >20 ppm, KH (carbonate hardness) 161.1 ppm, pH of 8.2, phosphate of >2 ppm, and DO_2_ of >90%. This falls within the typical temperature range of the natural habitat of *L. vannamei*, which is 26–32 °C, and corresponds to its natural salinity range of 15–35 [24].

### Photonic exposure

All animals were exposed to two 660 nm line lasers (LMI Gocator 2340 3R-R-01-T) (Table 1) under which they were passed at a speed of 6.7 cm/s, moved by a conveyor illuminated with background light of approximately 575 nm wavelength. The total exposure (rate of energy transfer per unit time) for one pass was 1.8 mW (1.8 milli-joules per second) (LMI pers com). This is below the exposure implicated in gross thermal damage in non-eye human tissue [24], though lower intensities are known to affect the vertebrate eye [25].

**Table 1:** Technical specifications for the LMI Gocator 2340 3R-R-01-T laser.

Model	2340
Data Points/Profile	1280
Linearity Z (+/- % of MR)	0.01
Resolution Z (mm)	0.013–0.037
Resolution X (mm) (Profile Data Interval)	0.095–0.170
Repeatability Z (μm)	1.2
Clearance Distance (CD) (mm)	190
Measurement Range (MR) (mm)	210
Field of View (FOV) (mm)	96–194
Laser Class	3R
Scan rate (Hz)	170–5000

Animals were alive during exposure and had been stored for a duration of at least 30 minutes in an illuminated room at ~1359 lux and wavelength of 579 nm prior to experimentation. Individual specimens were restrained by a Velcro strap over the back of the carapace, which secured the animal to a flat plastic tray and prevented tail-flicking and misalignment of the animal with the laser system or animal injury. As a control, all specimens were placed on the conveyor twenty times each consecutively, and the lasers were activated during some passes as set out in Table 2. The lights illuminating the conveyor were active during all passes.

**Table 2:** The number of individual prawns that were exposed to each of the five exposure levels.

Exposure (passes under the lasers)	Estimated maximum cumulative laser energy exposure (mW)	Prawn count
0	0	6
5	9	6
10	18	6
15	27	6
20	36	5

### Anaesthesia

While crustaceans were not initially protected in the UK as per the Animals (Scientific Procedures) Act (1986) [26], the recent Animal Welfare (Sentience) Act (2022) [5] extends the definition of sentience to include decapods.

Eugenol was chosen as an anaesthetic due to its efficacy in *Nephrops norvegicus* [27]. Eugenol served as the anaesthetic agent while ethanol facilitated its dispersal in the water. Dosage was calibrated for *L. vannamei* during an initial trial. Three animals were monitored with a Near Infrared Pulse Oximeter (SparkFun Pulse Oximeter and Heart Rate Sensor MAX32664 (Qwiic)) before and after exposure. Additionally, insensibility was tested by three tactile checks: limp limbs, no reaction upon touching the eye, and no motion in the maxillipeds. Heart rate measures were completed on a randomized selection of prawns throughout the experiment to ensure the consistency in the response of the animals to anaesthesia.

An anaesthetic bath was created using the calibrated dosage. The bath was heated to 27.0 ± 1°C and oxygenated with an air stone to minimise environmental distress and contained 1.5mL eugenol dissolved in 15 mL ethanol mixed into 5 L of seawater. After the completion of an experimental treatment, the animal was immediately placed in the anaesthetic bath and left submerged for at least twenty minutes. The animal was checked for insensibility using the responsiveness cues employed in the initial calibration. If the animal was not insensible after all three checks, the animal was replaced in the anaesthetic bath and reassessed after another ten minutes. Methods were designed with respect to the Animal Research: Reporting of *In Vivo* Experiments (ARRIVE) guidelines [28].

### Dissection and fixation

After being anaesthetised, the animals were removed from the bath, and the eyestalk was removed by severing it close to the base. One eye was taken from each prawn. The eye and eyestalk were then placed in an Eppendorf tube containing 10% neutrally buffered formalin, taken from a CellPath CellStor Pot (CellPath BAF-6000-08A), such that all tissues were covered. The animal was replaced into the eugenol to ensure continued insensibility in case any were not already euthanised. To ensure all animals were fully euthanised, they were finally placed in a plastic bag and frozen for at least 48 h.

### Sample processing

Sample processing was conducted in accordance with University of St Andrews School of Medicine Standard Operating Procedure - SASoM/METHOD/041.v6. Dehydration for embedding was performed by sequential infiltration of 70%, 80%, and 95% Industrial Methylated Spirits (IMS) for one hour at each concentration followed by 100% IMS for a total of 6 hours, then xylene for a total of 4.5 hours, all at 35 °C. Samples were then embedded in paraffin wax at 60°C. Prior to taking a section, the surface of the embedded sample was wiped with RDC (Rapid Decalcifier) to reduce the risk of tissue fragmentation. Two serial sections were cut along the sagittal plane of the eye with both the cornea and the eyestalk visible in one section. Sections taken from the same eye were mounted on the same slide.

All slides were stained with haematoxylin and eosin (HE). To prepare slides for staining, they were baked in an oven at 80 ° C for one hour, followed by sequential dewaxing in three wells of xylene for five minutes each, and rehydration in two wells of 100% alcohol, 80% alcohol, and 50% alcohol for two minutes each, after which slides were washed in running water for two minutes. Staining with HE was performed by immersion in haematoxylin for three minutes, followed by washing in running water for two minutes, and immersion in a well of Scott’s Tap Water Substitute until the tissue turned blue. After haematoxylin staining, the slide was placed in a well of Eosin Y aqueous solution for five minutes and was then washed in running water for two minutes. Staining with HE was followed by dehydration by sequential exposure to 50% and 80% alcohol for thirty seconds each, two wells of 100% alcohol for two minutes each, and three wells of xylene for five minutes each.

### Histology

Markers were quantified at each depth of the serially sectioned eye tissue, ranging between 18 and 50 sections, with a mean of 36 per eye. Markers were categorised based on an initial qualitative assessment; those which were poorly defined and rare, for example, highly variable in size and appearance, were quantified by coarse presence/ absence. Otherwise, markers were counted over a diagonal or ‘zigzag’ transect across the tissue section which was performed for all markers. Slides were viewed at 100 x magnification. Slides were viewed using an eyepiece reticule (Graticules Optics NE11A Indexed 10 x 10 grid, 1 mm pitch). The reticule overlaid the microscope field of view with a 10 x 10 grid consisting of 1 mm x 1 mm squares. The grid was indexed 1–10 horizontally and A through J vertically.

Counts were performed across one mounted level at a time in a transect (). The position of the grid was chosen by adjusting its position until only the bottom right corner of the grid appeared to contact any ommatidial or basement membrane (“valid”) tissue. A morphological feature at the bottom right corner was used as a landmark and the grid was moved until the landmark was contacted by the upper left corner. At this position, squares with features present would be counted using the counting protocol described below.

**Fig 2:**
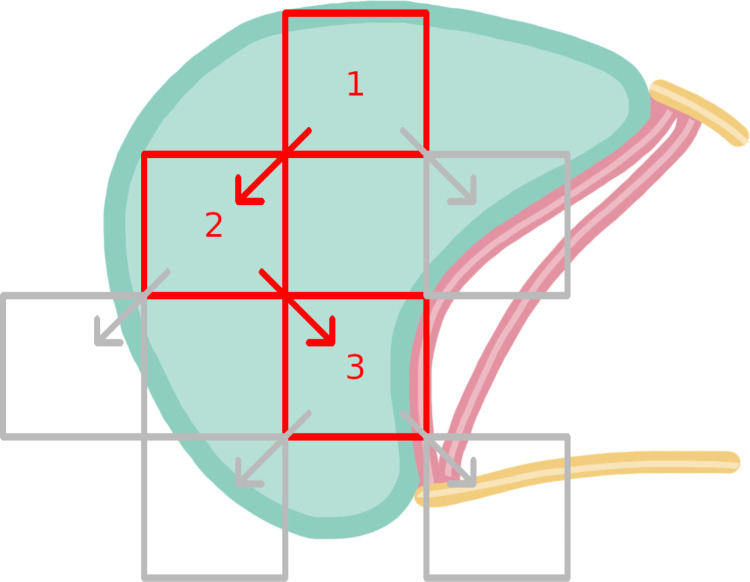
An illustration of a diagonal transect (red) and rejected grid positions (grey).

The starting position (1) is placed on the most extreme upper margin of the ommatidial layer (area filled in pale green). The next placement (2) is chosen as the usual direction of down and right would result in a position with <50% of the grid overlaying the ommatidial layer. The direction changes again for the same reasons. Position 3 is the final position as there are no downward directions which result in >50% of the grid overlaying the ommatidial layer.

Squares that did not contain any valid tissue were excluded and the number of these squares per section was recorded. The number of valid (tissue-containing) squares was defined as the total number of squares in the grid (100) minus the number of excluded squares. Squares to be analysed were selected by initial random number selection. Squares that had already been counted for that replicate and squares directly adjacent to squares that had already been counted, were excluded. This stratification method was chosen to address the possibility of selected squares being directly adjacent and therefore having similar traits (as per Dunnill [29]), as markers could appear in aggregations.

After the counting was completed at a given location, a landmark feature was identified at the bottom right corner of the grid and the grid was repositioned such that this landmark was in the top left corner. Subsequent repositions would be performed similarly until less than 50% of the grid contained valid tissue, after which the next repositioning would be performed using a landmark in the bottom left corner and repositioning it to the top right corner. If altering the direction of the diagonal transect did not amend the low presence of relevant tissue, the transect over that section was concluded.

### Ommatidial alteration

**Fig 3:**
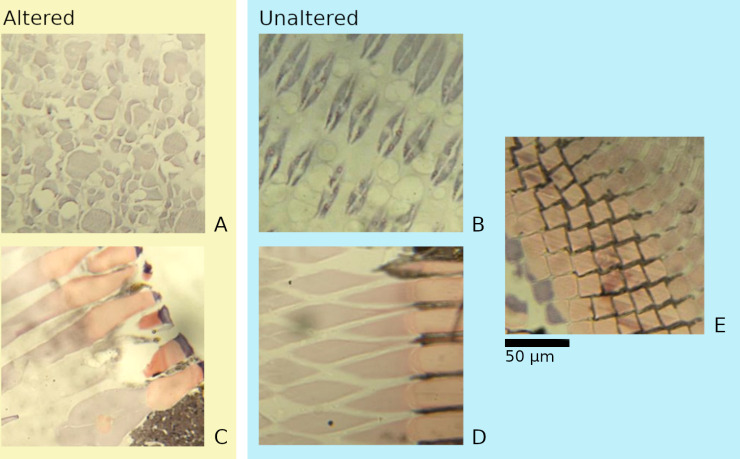
Various appearances of the ommatidial layer.

Objectively distinguishing between normally shaped and distorted ommatidia by eye proved challenging. This is due to the structure of the ommatidia, which in these prawns could be compared to a thin, hollow tube of cells which may connect at one end or another. A slice across an ommatidium would result in a conglomeration of cells in either a solid circle or in a ring with a hollow in the centre, while a slice along the longitudinal axis of an ommatidium would result in the appearance of two long parallel stripes of cells with varying degrees of separation. Further, the ommatidia naturally distort when exposed to light, making quantifications of how much damaging alteration was present impractical. Nevertheless, ommatidia which had been clearly distorted were distinct; additionally, cases in which the ommatidia had been destroyed or lost were observable (Fig 3). Due to these factors, changes to the ommatidia were split into two simple categories of ‘presence’ or ‘absence’ of alteration.

Altered (A and C) areas are distinct from primarily unaltered areas representing the same part of the eye (B and D, respectively) as well as generally well-ordered areas (E). Primarily unaltered areas (B, D, and E) are visually distinct from one another.

### Basement layer alteration

**Fig 4:**
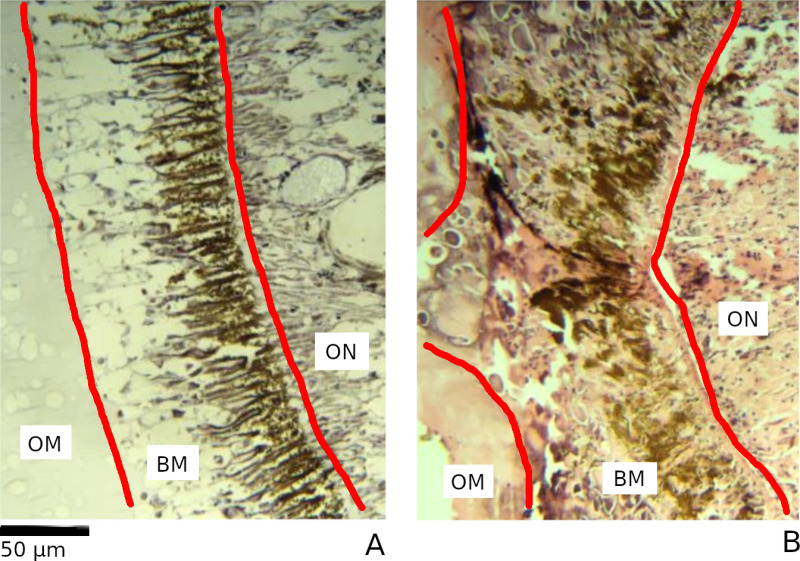
Unaltered (A) and altered (B) basement membrane in the eye of *L. vannamei.*

The basement layer separates the ommatidial layer from the optic nerve layer and contains high quantities of pigment as well as light-directing rhabdom, making alteration to this layer a potential indicator of photic damage. Alteration was described similarly to cases in *H. gammarus* [20–22]*,* with dissolution and disordering of the rhabdom across the membrane being a primary indicator (Fig 4). As with ommatidial distortion, there was wide variation in the appearance of both unaltered and altered basement layers. Alteration was therefore described as either present or absent for a given level.

The basement membrane (BM) is positioned between the ommatidial layer (OM) and the optic nerve layer (ON). These layers are divided with red lines for clarity. The unaltered basement membrane has the appearance of orderly “stripes” of pale coloured rhabdom across the brown melanin from the ommatidial layer to the optic nerve layer. The altered basement membrane loses this pattern and instead has a disordered appearance with no clear rhabdom structures.

### Melanin

**Fig 5:**
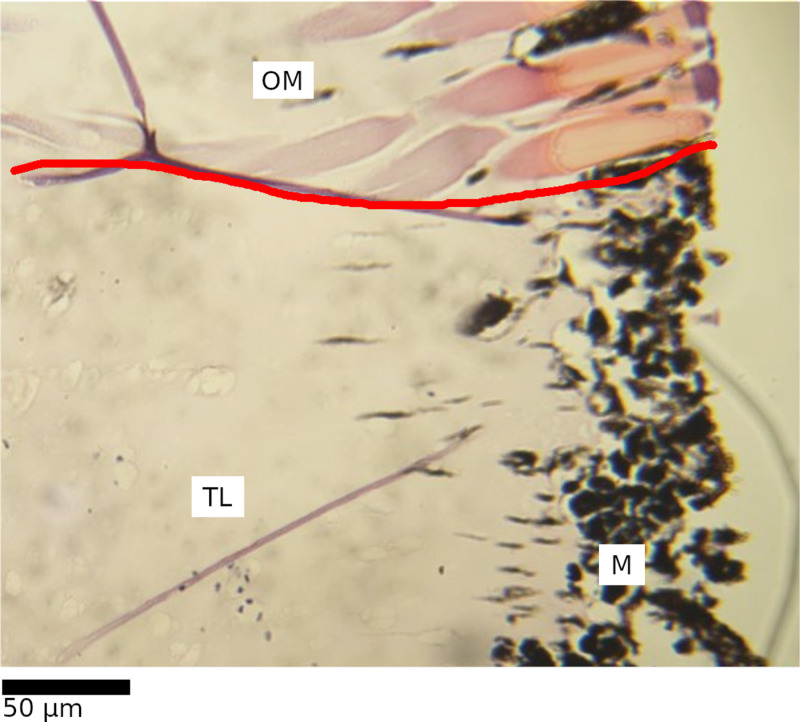
Melanin-associated tissue alteration.

Melanin-associated tissue alteration was considered as a metric due to the absorptive nature of shielding pigments resulting in high concentrations of photic energy in these pigments. Melanin-associated tissue alteration was evaluated as either present or absent. The appearance of alteration of this type was defined by the presence of large concentrations of melanin in areas in which melanin would be expected (near, and in, the basement membrane, and near the exterior of the eye) containing no cells typically expected in that area (Fig 5). The necessity of a high melanin presence was used to distinguish melanin-associated alteration from artefacts, which would contain no or minimal debris.

**Fig 6:**
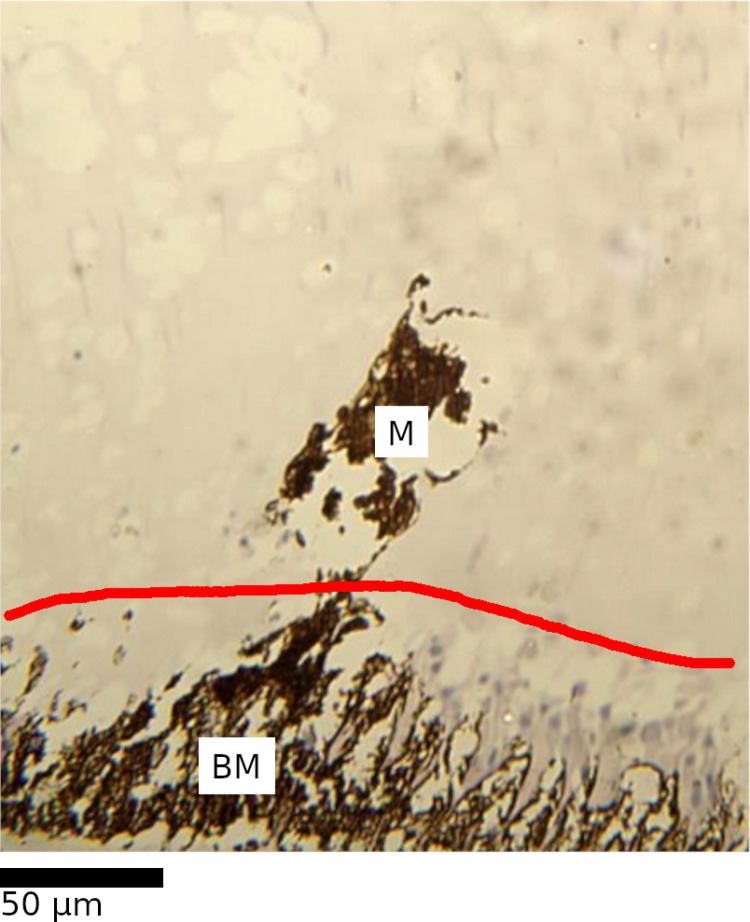
Melanin clump.

Melanin clumping was quantified by stratified random sampling of data across a diagonal or “zigzag” transect. Melanin clumps were defined as areas with high concentrations of melanin that were not within the bounds of the basement layer, and that were not confined to the spaces between otherwise healthy ommatidial subunits near the exterior of the eye (Figs 5, 6). The degree of melanin clumping was expressed as the proportion of sampled squares over a transect which contained melanin clumps.

The alteration appears as clumped melanin (M) exterior to an area in which usual eye structures are not present (TL). Expected eye structures (OM) of the ommatidial layer are immediately adjacent to the sites of alteration, with distinct “borders” demarcated by red lines for clarity.

The melanin clump (M) extends out of an altered basement membrane (BM), where melanin presence is expected, into the central ommatidial layer, where high melanin presence is not typical. The qualitatively estimated boundary, based on the extent of the “light purple” intact basement membrane components adjacent to the pigment, between the basement membrane and the ommatidial layer is marked with a red line.

### Haemocyte infiltration

**Fig 7:**
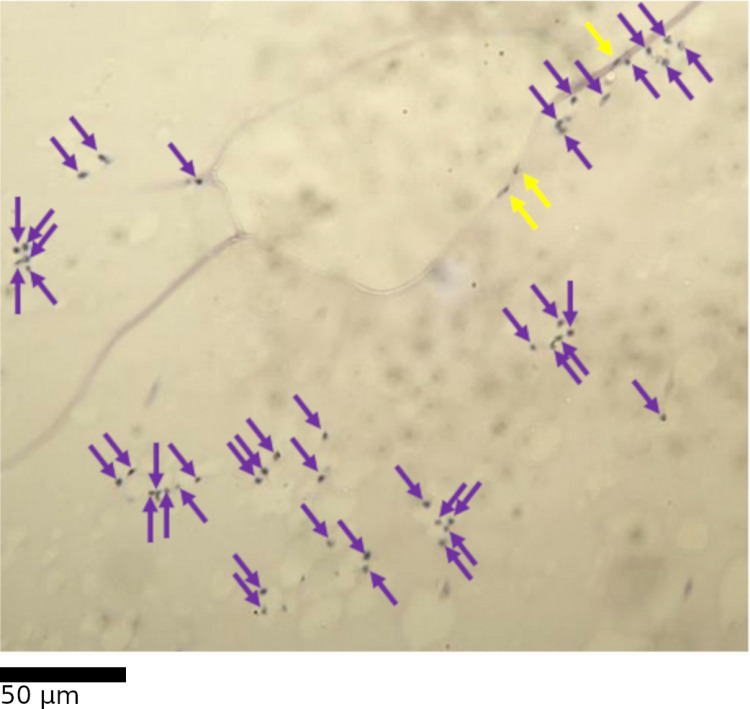
Evidence of haemocyte infiltration into the ommatidial layer.

Haemocyte presence in the ommatidial layer does not immediately suggest damage, but haemocyte presence is generally low in healthy eyes [20] and heightened presence may be indicative of a ruptured basement membrane. Haemocytes were defined as dark, singular cells, sometimes with a visual definition between the nucleus and the rest of the cell (Fig 7).

Haemocytes are marked with purple arrows. Unconfirmed features which may or may not be haemocytes are marked with yellow arrows.

Simple presence or absence of haemocytes was considered too coarse a metric, and instead haemocytes were quantified using the diagonal transect. Individual haemocytes were counted in squares selected for inclusion in the stratified random sample. Haemocyte count was expressed as haemocytes-per-square, for all squares sampled over a transect.

### Lesions

**Fig 8:**
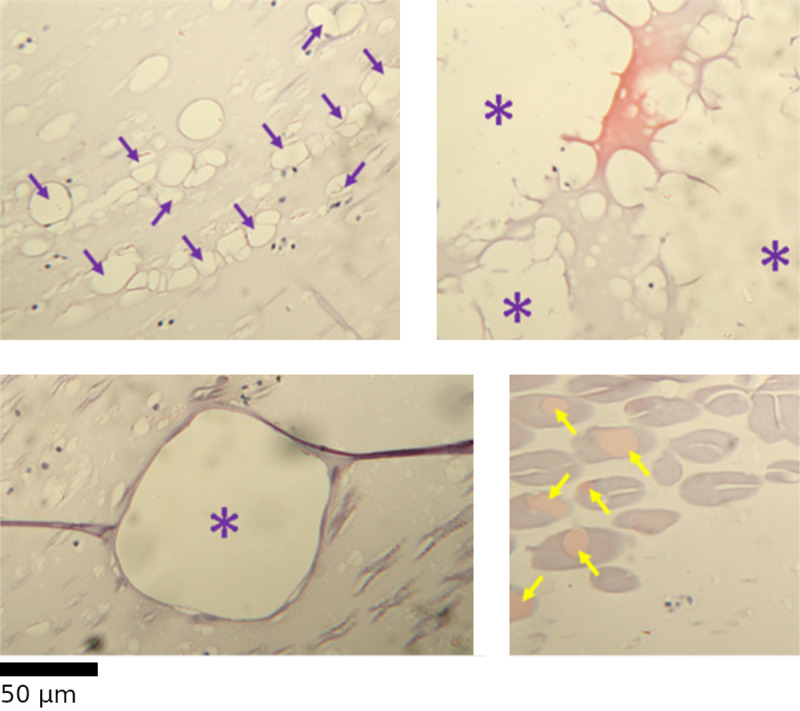
Various appearances of lesions.

Lesions were variable in appearance, size, and location. There were two major types of appearances: lesions appearing as an area of total tissue loss and those that were eosinophilic (“pink”) and rounded in shape (Fig 8). Loss lesions were very numerous and were counted using the diagonal transect, and it was important to distinguish between true lesions and non-photic tissue-loss artefacts. The presence of melanin clumping around the edge of the zone of loss was one indication of lesion-associated loss. In most cases, the edge would appear visually darker than the unaffected tissue, even without melanin present. Lesions could appear in large aggregations, resulting in a dispersed, disordered tissue appearance. Additionally, if tissue loss was over a contiguous area that connected to the space surrounding the mounted level, it would be more appropriately described as an artefact such as a tear. Tears would occasionally have a thick dark area near one edge, the result of the torn tissue folding over itself.

Eosinophilic lesions were distinguished from artefacts such as colour distortion and occlusion by tissue fragments using a different set of indicators. They must present as an interruption in usual tissue appearance, for example, a rounded unit within the angular photo-directive layer. They must not overlay other features because appearing “in front of” other features is indicative of the eosinophilic colour resulting from an overlying tissue fragment.

Loss lesions (purple indicators) can appear “smooth” or “ragged” as well as “contiguous” or “clusters” of smaller lesions. Eosinophilic lesions (yellow arrows) appear as rounded, eosinophilic (“pink“) intrusions into the tissue where this colour is not expected.

Individual lesions were not counted due to the high variability in size and severity; instead, a given grid square would be marked as lesioned or not, similar to the pigment clump presence/absence. Lesion presence was expressed as the proportion of grid squares with lesions present.

### Optic nerve fibre necrosis

**Fig 9:**
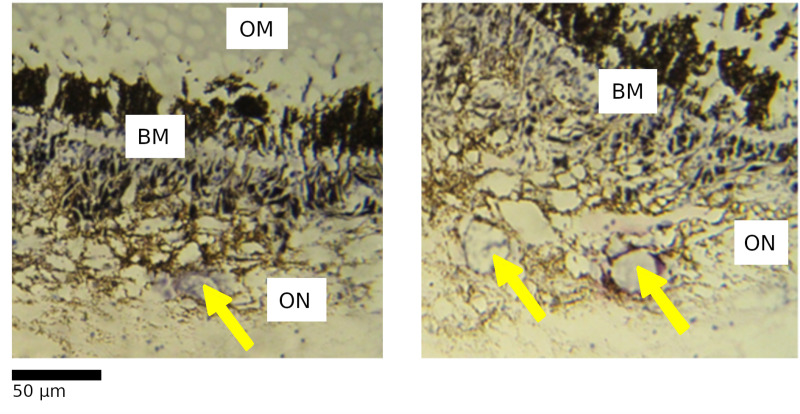
The location and appearance of optic nerve fibre necrosis.

Optic nerve fibre (ONF) necrosis was manually evaluated. The ONF layer was present directly behind the basement membrane; necrosis elsewhere was not quantified. Necrosis was defined as localised units of dispersed, unordered, basophilic fibres with a visually distinct boundary against healthy nerve fibres and no nuclei (Fig 9) [20]. Necrosis manifested inconsistently, in “patches” of varied size, shape, and connectedness. Due to this high variability in appearance, ONF was quantified broadly as either present or absent.

Necrotic areas (yellow arrow) appeared as basophilic (purple) areas with a “diffused” or “cloudy” appearance, disordered structure, and a lack of nuclei present within the optic nerve fibre layer (ON) directly behind the basement membrane (BM).

### Histological artefacts

In most cases, slides had tissue alterations that were likely to be artefacts. Artefact presence and type was recorded for each sample, with types including folding, tearing, and occlusion by objects such as bubbles or foreign particles. The different rates of dehydration-associated shrinkage of tissue caused large empty areas to appear between the edge of the ommatidial layer and the outer extreme of the eye, but the occurrence of this was not catalogued as this area was not relevant to any markers. Folding of the tissue on the slide could result in the top layer of the fold obscuring the appearance of the layer below. Tissue loss in any location could result in features such as lesions and pigment clumps being missed. Where tissue loss was severe enough to render the evaluation of certain markers impossible, this was denoted as extreme tissue loss. Cases in which certain markers could not be quantified due to non-artefactual causes, such as level depth being too shallow to view the photoreceptive layer, were not considered to be cases of extreme tissue loss.

### Data analysis

Data (S1 Table ) were analysed using R Statistical Software [30]. Mixed models were used to account for within-sample variability across exposure levels and were chosen for their suitability in handling hierarchical data, where eye tissue levels from the same sample might show correlated responses. The lme4 package [31] was used to fit these models, with the tissue level defined as a random effect to account for repeated measurements within individual crustaceans. Akaike’s information criterion (AIC) and a likelihood ratio test (LRT) were used to compare models and to assess the statistical significance of exposure level as a predictive variable for each response marker. ΔLogLik is the change in log likelihood between models fit with and without the laser exposure as a predictive variable. To validate model assumptions, we used the DHARMa package [32] to check for the normality of residuals and homoscedasticity. Tissue levels with extreme data loss (39 cases) were excluded from the analysis to ensure accuracy and consistency across models.

## Results

The effects of laser exposure on specific ocular tissue markers in *L. vannamei* are assessed for each marker’s response across varying exposure levels. By applying mixed models to account for intra-sample variability, we evaluated the likelihood of changes in presence/absence markers and measured counts or proportions for transect markers. Results for each marker are presented in two categories: presence/absence markers (ommatidial layer alteration, basement membrane alteration, melanin-associated tissue alteration, and optic nerve fibre necrosis) and transect markers (melanin clumping, haemocyte count, and lesion incidence).

### Presence/absence markers

**Fig 10:**
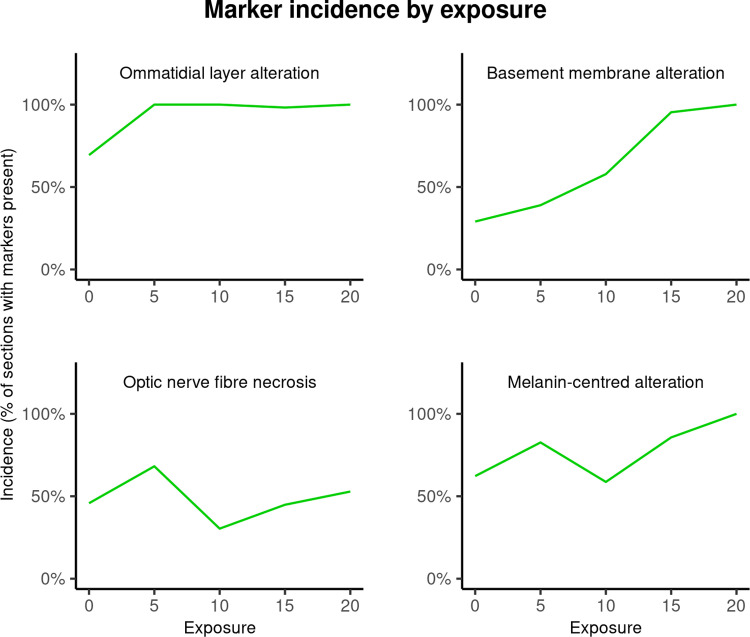
Presence/absence marker incidence by exposure.

Presence/absence markers include ommatidial layer alteration, basement membrane alteration, melanin-associated tissue alteration, and optic nerve fibre necrosis. Of these, basement membrane alteration (Chisq = 22.97, Df = 1, ΔAIC = 20.96, ΔLogLik = 11.49, P < 0.001) and melanin-associated tissue alteration (Chisq = 4.09, Df = 1, ΔAIC = 2.09, ΔLogLik = 2.04, P = 0.04) were significantly and positively correlated with increases in exposure level (Fig 10). Conversely, there was no strong evidence that the incidence of ommatidial layer alteration (Chisq = 0.62, Df = 1, ΔAIC = 1.38, ΔLogLik = 0.31, P = 0.43) or optic nerve fibre necrosis (Chisq = 0.01, Df = 1, ΔAIC = 1.99, ΔLogLik = 0, P = 0.92) were correlated with exposure level (Fig 10).

Incidence (% of tissue levels with a marker present) of basement membrane (BM) and ommatidial layer (OL) alteration, optic nerve fibre (ONF) necrosis, and melanin-associated (MA) alteration, by exposure level. BM (Chisq = 22.97, Df = 1, ΔAIC = 20.96, ΔLogLik = 11.49, P < 0.001) and MA alteration (Chisq = 4.09, Df = 1, ΔAIC = 2.09, ΔLogLik = 2.04, P = 0.04) were significantly positively correlated with increases in exposure level. There was no strong evidence that the incidence of OL alteration (Chisq = 0.62, Df = 1, ΔAIC = 1.38, ΔLogLik = 0.31, P = 0.43) or ONF necrosis (Chisq = 0.01, Df = 1, ΔAIC = 1.99, ΔLogLik = 0, P = 0.92) were correlated with exposure level.

### Transect markers

**Fig 11:**
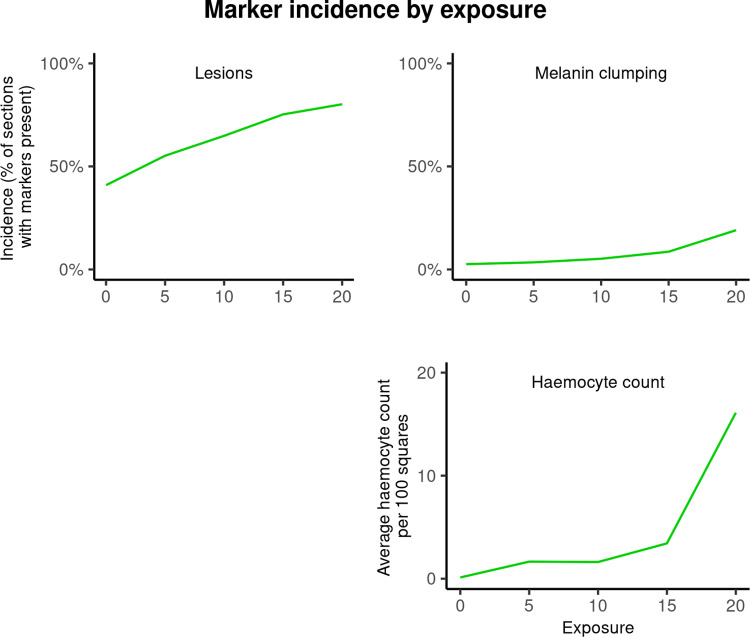
Transect marker incidence by exposure.

Transect markers include melanin clumping incidence (Chisq = 16.62, Df = 1, ΔAIC = 15.4, ΔLogLik = 8.3, P < 0.001), haemocyte count (Chisq = 12.35, Df = 1, ΔAIC = 10.36, ΔLogLik = 6.17, P < 0.001), and lesion incidence (Chisq = 16.67, Df = 1, ΔAIC = 14.7, ΔLogLik = 8.3, P < 0.001). All these markers were significantly positively correlated with increasing exposure level (Fig 11).

Incidence (% of sampled squares with a marker) of melanin clumping and lesions, and haemocyte count (average haemocytes per 100 squares), by exposure level. Melanin clumping (Chisq = 16.62, Df = 1, ΔAIC = 15.4, ΔLogLik = 8.3, P < 0.001), lesioning (Chisq = 16.67, Df = 1, ΔAIC = 14.7, ΔLogLik = 8.3, P < 0.001), and haemocyte count (Chisq = 12.35, Df = 1, ΔAIC = 10.36, ΔLogLik = 6.17, P < 0.001) were all significantly positively correlated with increases in exposure level.

### Eugenol anaesthesia

The heart rate of prawns was observed to be approximately 120 beats per minute before exposure to eugenol. This was observed to be approximately 80 beats per minute after exposure, suggesting a lowered activity state. Furthermore, there was a lack of responsiveness cues indicating insensibility occurred within 5–15 minutes.

## Discussion

Novel imaging techniques are increasingly being used in combination with machine learning to automate data acquisition from biological systems and in challenging environments. The semi-automated Plankton Imager has demonstrated the ability to capture temporal variations in mesozooplankton communities with high spatial and temporal resolution [33]. Similarly, advancements in underwater computational imaging have overcome the challenges of light attenuation and scattering, offering solutions to enhance imaging quality and resolution in aquatic environments [34]. Novel technologies offer opportunities to address data deficiencies in areas such as habitat and stock assessments, providing tools for ecosystem monitoring and sustainable fisheries management. The potential impact that these technologies may have on their respective target species or environments has attracted little attention.

This study represents the first assessment of the potential impact of a 3D laser scanning device, the CatchS3ID, on the eyes of a crustacean, thus helping to establish a baseline and methodology for future studies with other potentially sensitive crustacean species. To exploit the potential of a range of imaging technologies for data collection from living animals, particularly those regarded as legally “sentient”, further research is required to ensure that the welfare and survival of scanned animals is not compromised. The CatchS3ID could transform how stock assessment data for crustaceans and potentially other commercially important species is acquired. Widespread acceptance and adoption of this technology could lead to more robust, regionally meaningful, and dynamic stock assessment, supporting more effective fisheries management and sustainable exploitation of stocks. The automation of data acquisition and engaging fishers in this process may also reduce the cost of stock assessment, and encourage greater transparency and co-management of fisheries. However, welfare and survival of animals scanned by the CatchS3ID must be assured before deployment.

The incidence rate of four of the six selected markers was significantly and positively correlated with increasing exposures to the laser. This suggests that repeated exposures to the laser scanner may cause cumulative ocular tissue damage in *L. vannamei*. Ommatidial layer alteration, optical nerve fibre necrosis, lesions and melanin-centred alteration were all present before exposure to the laser. There are several potential explanations for these observations. Exposure to ambient light levels may be sufficient to induce changes to these tissues and for ommatidial layer alteration levels of 100% means any possible effects from laser exposure are not detectable. Ambient light levels are shown to induce changes in ocular tissues in some crustaceans, such as *Nephrops norvegicus*, which are habituated to darker environments [14,16,34]. The likelihood of similar damage by ambient light levels in *L. vannamei* may be assessed by comparing the habitats of *L. vannamei* to *N. norvegicus*. Unlike *N. norvegicus, L. vannamei* typically resides in much shallower habitats such as estuaries, coastal waters, and salt marshes, in tropical climates [24]. Both shallowness and lower latitudes suggest that *L. vannamei* may experience higher irradiance in its natural habitat [35]. Should susceptibility to changes in ocular tissue at ambient light levels be related to the light levels the animal is typically exposed to, *L. vannamei* may be more resistant to such changes. Relatively more resistance does not exclude the possibility of ambient light levels inducing changes in the animals in this study, but with this difference in resistance in consideration, other factors should be considered.

Additionally, the animals used in this study were somewhat senescent for this species, and whilst there was no indication of injury or disease in them, their advanced age may be reflected in age-related tissue degradation amongst other influential factors. Senescence in crustaceans varies by species in speed and in the pattern of degeneration, for example, the cessation of moulting alongside the cessation of regeneration and detoxification of tissues [36]. In captivity, this age-related degeneration becomes the primary cause of death [36] and therefore may be a more important consideration for these individuals reaching end-of-life in captivity. The susceptibility to photothermal damage may also be a function of age and may warrant separate investigation. Furthermore, diseased tissue areas in crustaceans are typically encapsulated by a melanin layer and then destroyed, so an increase in age-related disease may induce changes in the appearance of melanin in tissue [36]. If diseased tissue developed in the eyes of the tested individuals, a baseline level of melanin distortion may be expected even without any photothermal damage. Raised baseline incidence of markers may also obscure effects, as incidence rates could not exceed 100% for all markers except haemocytes. The effects of senescence on the utility of ocular tissue changes would need to be evaluated at the species level, or at least in crustacean species with different patterns and speeds of tissue degradation. For example, the relatively short-lived *L. vannamei* may experience greater age-related changes than longer-lived cold-water species that are considered to have “negligible” degradation [36]. For the purposes of this study, all the animals were of similar age, and thus while senescence may have influenced the presence of ocular changes overall, it is unlikely that there was a substantial variation in age-related degradation between individuals. Melanin-associated tissue alteration, basement membrane alteration, lesions, melanin clumping, and haemocyte count all increased linearly between 15% and 25% change with repeated laser exposure. Given a baseline of zero for melanin clumping and haemocyte count followed by a gradual increase with repeated laser exposures, these indicators may be useful as an indication of response to laser exposure being less confounded by ambient light level or possible age-related tissue damage.

The intensities of laser exposure used in this research were selected based on the identification of the worst-case scenario in terms of dose. Anticipated use of the device would require that the specimen is scanned once and at a conveyor speed in the order of 25 cm/s (4 times faster) than used in the experiments. Assuming that the laser line scanning rate remains the same, increasing the conveyor speed reduces the exposure of the eye to laser energy. The 660 nm 3R lasers used in this experiment deliver ~1.8 mW (1.8 milli-joules per second) based on an aperture size of 7 mm * 7 mm (LMI, pers com.) at 225 mm between the laser source and the specimen. At a speed of 6.7 cm/s suggests that an aperture of 7 mm * 7 mm (a larger cross-sectional area than any of the crustacean eyes scanned) the energy exposure would equate to 0.19 mW. At an operational conveyor speed that is four times faster, this energy exposure would reduce to ~ 0.05 mW.

Incidental observation of the behaviour of animals suggested that a single scan has no discernible effect on the ability of the specimen to exhibit normal cryptic behaviour, seeking out darkness. Further experimentation is required using other commercially fished crustaceans, the European lobster *Homerus gamarus* and brown crab *Cancer pagurus* in particular. The results of this research may inform future experiments, providing a framework for semi-quantitative histological analysis of the implications of using 3R lasers to produce 3D point cloud images for morphometric analysis. Immunochemical histological analysis may provide additional resolution.

Biochemical reactions can also serve as indicators of eye damage. Free radicals, including reactive oxygen species (ROS), are oxidants produced by normal aerobic metabolism [11]. In addition to ROS production caused by regular metabolic processes, external sources such as light can contribute to increased ROS [7,34,37–40]. Cellular and tissue components can be damaged by excess ROS, negatively affecting vision. Senescence may also affect levels of ROS as senescent animals may have reduced detoxification of tissues leading to a raised baseline presence of ROS or even an accumulation of ROS over time [36]. For any future investigation of ROS as a marker of photothermal damage to the eye, the age and species of the animals being studied should be considered.

To best inform the future development of live-animal laser scanners for fisheries, further investigation into the morphological impacts of only one exposure is needed. However, the outcomes of this study may suggest that operating procedures for the scanner, or similar ones, should stipulate that animals should not be scanned more than once. Additionally, the significant positive relationship between exposure and basement membrane alteration, melanin-associated tissue alteration, haemocyte presence, lesion incidence, or pigment clumping incidence suggests that these markers may be used in future studies to evaluate photothermal damage using light microscopy.

## Conclusion

The use of 660nm diode lasers as described in this paper indicates that multiple exposures can result in ocular tissue damage in *L. vannamei*. However, the exact nature of this damage is difficult to define because the age of the experimental animals could also have resulted in natural chronic tissue degradation. In an operational context, where the CatchS3ID is being used to collect morphometric data for stock assessment purposes, each animal will only be subject to a single laser exposure and pass through the laser at a speed approximately 4 times faster than used in these experiments. The laser energy exposure is therefore likely to be ~ 0.05 mW and unlikely to induce significant ocular tissue damage. Whilst the eye structure of *L. vannamei* represents a reasonable analogue of commercially fished crabs and lobsters, further research focusing on the target species for the fisheries in which the CatchS3ID is likely to be deployed is required. The discarding of live crustaceans is largely accepted on the basis that their subsequent post-capture survival is unimpaired by the process of capture, at least to the extent that it is not considered to result in levels of mortality likely to affect the survival of the population. The impact of ambient light exposure on crustacean eyes is largely unknown and therefore the implications for accepted fishing practices remain opaque with respect to welfare considerations.

## Supporting information

S1 Table Experimental data on laser exposure and associated ocular tissue changes in *L. vannamei.* Observations of tissue alterations across varying exposure levels and replicates.
(XLSX)

## References

[1] Gebremedhin S, Bruneel S, Getahun A, Anteneh W, Goethals P. Scientific methods to understand fish population dynamics and support sustainable fisheries management. Water. 2021;13(4):574. doi: 10.3390/w13040574

[2] Hilborn R, Ovando D. Reflections on the success of traditional fisheries management. ICES J Mar Sci. 2014;71(5):1040–6. doi: 10.1093/icesjms/fsu034

[3] Mesquita C, Miethe T, Dobby H, Mclay A. Crab and lobster fisheries in Scotland: results of stock assessments 2013–2015. Scott Mar Freshw Sci. 2017;8(14):1990–1.

[4] Mesquita C., Ellis A., Miethe T., Dobby H. Crab and lobster fisheries in scotland: results of stock assessments 2016-2019. Scott Mar Freshw Sci 2023;14(5).

[5] Animal Welfare (Sentience) Act. UK Public General Acts; 2022 [updated 28 April 2022; cited 27 Oct 2022]. Available from: https://www.legislation.gov.uk/ukpga/2022/22/enacted

[6] Cios A, Cieplak M, Szymański Ł, Lewicka A, Cierniak S, Stankiewicz W, et al. Effect of different wavelengths of laser irradiation on the skin cells. Int J Mol Sci. 2021;22(5):2437. doi: 10.3390/ijms22052437 33670977 PMC7957604

[7] Huang C, Wang JJ, Ma JH, Jin C, Yu Q, Zhang SX. Activation of the UPR protects against cigarette smoke-induced RPE apoptosis through up-regulation of Nrf2. J Biol Chem. 2015;290(9):5367–80. doi: 10.1074/jbc.M114.603738 25568320 PMC4342454

[8] Huang Z, He T, Zhang J, Du C. Red light irradiation as an intervention for myopia. Indian J Ophthalmol. 2022;70(9):3198–201. doi: 10.4103/ijo.IJO_15_22 36018087 PMC9675534

[9] Cronin TW. Optical design and evolutionary adaptation in crustacean compound eyes. J Crustacean Biol. 1986;6(1):1. doi: 10.2307/1547926

[10] Meyer-Rochow VB. The crustacean eye: dark/light adaptation, polarization sensitivity, flicker fusion frequency, and photoreceptor damage. Zoolog Sci. 2001;18(9):1175–97. doi: 10.2108/zsj.18.1175 11911074

[11] Bryceson KP, McIntyre P. Image quality and acceptance angle in a reflecting superposition eye. J Comp Physiol. 1983;151(3):367–80. doi: 10.1007/bf00623912

[12] Kleinlogel S, White AG. The secret world of shrimps: polarisation vision at its best. PLoS One. 2008;3(5):e2190. doi: 10.1371/journal.pone.0002190 18478095 PMC2377063

[13] Nilsson HL, Lindström M. Retinal damage and sensitivity loss of a light-sensitive crustacean compound eye (*Cirolana Borealis*): electron microscopy and electrophysiology. J Exp Biol. 1983;107(1):277–92. doi: 10.1242/jeb.107.1.277

[14] Chapman CJ, Shelton PMJ, Shanks AM, Gaten E. Survival and growth of the Norway lobster *Nephrops norvegicus* in relation to light-induced eye damage. Mar Biol. 2000;136(2):233–41. doi: 10.1007/s002270050681

[15] Gaten E, Moss S, Johnson ML. The reniform reflecting superposition compound eyes of *Nephrops norvegicus*: optics, susceptibility to light-induced damage, electrophysiology and a ray tracing model. Adv Mar Biol. 2013;64:107–48. doi: 10.1016/B978-0-12-410466-2.00004-2 23668589

[16] Shelton PM, Gaten E, Chapman CJ. Light and retinal damage in *Nephrops norvegicus* (L.) (Crustacea). Proc R Soc B 1985;226(1243):217–36.

[17] Viljanen MLM, Nevala NE, Calais-Granö CL, Lindström KMW, Donner K. Increasing the illumination slowly over several weeks protects against light damage in the eyes of the crustacean *Mysis relicta*. J Exp Biol. 2017;220(Pt 15):2798–808. doi: 10.1242/jeb.155101 28515237

[18] Wu J, Seregard S, Algvere PV. Photochemical damage of the retina. Surv Ophthalmol. 2006;51(5):461–81. doi: 10.1016/j.survophthal.2006.06.009 16950247

[19] Meyer-Rochow VB. Light-induced damage to photoreceptors of spiny lobsters and other crustaceans. Crustac. 1994;67(1):95–109. doi: 10.1163/156854094x00332

[20] Maniscalco AM, Shields JD. Histopathology of idiopathic lesions in the eyes of *Homarus americanus* from Long Island Sound. J Invertebr Pathol. 2006;91(2):88–97. doi: 10.1016/j.jip.2005.09.007 16376928

[21] Magel CR, Shields JD, Brill RW. Idiopathic lesions and visual deficits in the american lobster (*Homarus americanus*) from Long Island Sound, NY. Biol Bull. 2009;217(1):95–101. doi: 10.1086/BBLv217n1p95 19679726

[22] Shields JD, Wheeler KN, Moss JA. Histological assessment of the lobster (*Homarus americanus*) in the “100 Lobsters” project. J Shellfish Res. 2012;31(2):439–47. doi: 10.2983/035.031.0204

[23] Treece GD, Fox JM. Design, operation and training manual for an intensive culture shrimp hatchery (with emphasis on *Penaeus monodon* and *Penaeus vannamei*). NOAA Institutional Respository, TAMU-SG 1998; 93–505. Available from: https://repository.library.noaa.gov/view/noaa/12406

[24] Briggs M, Fox J. Litopenaeus vannamei (whiteleg shrimp). CABI Compendium; 2007.

[25] Schulmeister K, Jean M. The risk of retinal injury from Class 2 and visible Class 3R lasers, including medical laser aiming beams. Med Laser Appl. 2010;25(2):99–110. doi: 10.1016/j.mla.2010.01.005

[26] Animals (Scientific Procedures) Act. UK Government Animals in Science Committee; 1986 [updated 14 June 2017; cited 27 Oct 2022]. Available from: https://www.gov.uk/government/publications/consolidated-version-of-aspa-1986.

[27] Spoors F, James MA, Mendo T, McKnight JC, Bønnelycke E-MS, Khan N. Investigating clove oil and its derivatives as anaesthetic agents for decapod crustaceans to improve welfare commercially and at slaughter. Front Anim Sci. 2023;4. doi: 10.3389/fanim.2023.1180977

[28] Percie du Sert N, Hurst V, Ahluwalia A, Alam S, Avey MT, Baker M. The ARRIVE guidelines 2.0: updated guidelines for reporting animal research. PLoS Biology. 2020;18(7):e3000410. doi: 10.1371/journal.pbio.3000410 32663219 PMC7360023

[29] Dunnill MS. Evaluation of a simple method of sampling the lung for quantitative histological analysis. Thorax. 1964;19(5):443–8. doi: 10.1136/thx.19.5.443 14216974 PMC1018858

[30] R Core Team. R: A language and environment for statistical computing. R Foundation for Statistical Computing, Vienna, Austria 2022. Available from: https://www.R-project.org/.

[31] Bates D, Mächler M, Bolker B, Walker S. Fitting linear mixed-effects models Usinglme4. J Stat Soft. 2015;67(1). doi: 10.18637/jss.v067.i01

[32] Hartig F. DHARMa: Residual Diagnostics for Hierarchical (Multi-Level/ Mixed) Regression Models. R package version 0.4.6 2022. Available from: https://CRAN.R-project.org/package=DHARMa.

[33] Scott J, Pitois S, Close H, Almeida N, Culverhouse P, Tilbury J, et al. In situ automated imaging, using the Plankton Imager, captures temporal variations in mesozooplankton using the Celtic Sea as a case study. J Plankton Res. 2021;43(2):300–13. doi: 10.1093/plankt/fbab018

[34] Fletcher AE. Free radicals, antioxidants and eye diseases: evidence from epidemiological studies on cataract and age-related macular degeneration. Ophthalmic Res. 2010;44(3):191–8. doi: 10.1159/000316476 20829643

[35] Lewis WM Jr. Tropical lakes: how latitude makes a difference. Perspect Trop Limnol. 1996;12(5):43–64.

[36] Vogt G. Growing old: aging in crustacea. In: Life Histories. Wellborn GA, Thiel M, editors. Oxford University Press: 2018.

[37] Chan C-M, Huang C-H, Li H-J, Hsiao C-Y, Su C-C, Lee P-L, et al. Protective effects of resveratrol against UVA-induced damage in ARPE19 cells. Int J Mol Sci. 2015;16(3):5789–802. doi: 10.3390/ijms16035789 25775159 PMC4394506

[38] Higuchi A, Ito K, Dogru M, Kitamura M, Mitani F, Kawakita T, et al. Corneal damage and lacrimal gland dysfunction in a smoking rat model. Free Radic Biol Med. 2011;51(12):2210–6. doi: 10.1016/j.freeradbiomed.2011.09.025 22001743

[39] Saccà SC, Roszkowska AM, Izzotti A. Environmental light and endogenous antioxidants as the main determinants of non-cancer ocular diseases. Mutat Res. 2013;752(2):153–71. doi: 10.1016/j.mrrev.2013.01.001 23337404

[40] Wu X, Cobbina SJ, Mao G, Xu H, Zhang Z, Yang L. A review of toxicity and mechanisms of individual and mixtures of heavy metals in the environment. Environ Sci Pollut Res Int. 2016;23(9):8244–59. doi: 10.1007/s11356-016-6333-x 26965280

