# Correction: Assessing the effects of a 660 nm diode laser on crustacean eyes

**DOI:** 10.1371/journal.pone.0349039

**Published:** 2026-05-07

**Authors:** Rhys A. C. Hague, James E. V. Rimmer, Mark A. James

The following information is missing from the Funding statement: Views and opinions expressed are however those of the author(s) only and do not necessarily reflect those of the European Union or REA. Neither the European Union nor the granting authority can be held responsible for them.
